# Teaching Academic Staff to Implement Interactive Graphics for Their Courses

**DOI:** 10.1007/s10758-023-09652-y

**Published:** 2023-05-31

**Authors:** Pamela Liebig, Viviane Filor, Mariana Scheumann, Martina Buchholz, Klaus Jung

**Affiliations:** 1 Institute for Animal Breeding and Genetics, University of Veterinary Medicine Hannover, Foundation, Bünteweg 17p, 30559 Hannover, Germany; 2 Department of Pharmacology, Toxicology and Pharmacy, University of Veterinary Medicine Hannover, Foundation, Hannover, Germany; 3 Institute of Zoology, University of Veterinary Medicine Hannover, Foundation, Hannover, Germany; 4 Institute for Food Toxicology, University of Veterinary Medicine Hannover, Foundation, Hannover, Germany

**Keywords:** Interactive graphics, Professional development, R-programming, R-shiny, Workshop

## Abstract

Many academic teachers present data as static graphics in their lectures and courses. However, data structures have become more complex in the last decades, especially in the biomedical disciplines, and interactive graphics can provide a better means to communicate the scientific contents to students. Besides, the technological qualifications of academic staff are diverse, and it has been little studied how these teachers can be trained to program and implement interactive graphics for their courses. It is also little known whether interactive graphics will be helpful for teaching and learning. We conducted a pilot online workshop to identify aspects that need to be addressed when teaching academic staff to program interactive graphics in form of Shiny-apps based on the programing language R. The n = 25 participants were academic staff from the fields of medicine or natural sciences. Pre- and post-workshop questionnaires were used to identify which aspects should be considered in future workshops, to query the usefulness of interactive graphics for teachers and students, and to identify the impact of the workshop on the teacher’s opinion towards digital teaching devices and their preparedness to program own interactive graphics. Most participants showed strong interest to use interactive graphics in their courses after the workshop, and interactive graphics were overall rated as very helpful for teachers and students. However, only those with prior programming knowledge intended to implement own graphics. We conclude that an extension of the workshop will be necessary to provide additional training for participants with no background in R programming.

**Supplementary Information:**

The online version contains supplementary material available at 10.1007/s10758-023-09652-y.

## Introduction

Graphics are widely used in academic teaching to visualize correlations, patterns, and other information from scientific data in general. Typically, these graphics are presented as a static image in a wall presentation. However, many scientific data that is collected in studies and experiments nowadays is very complex, particularly in biomedical research, and a static graphic may not be sufficient to transport all the information contained in a data set. Aside, the advances of computer and network technologies have added new ways for data presentation. Various visualizations tools have emerged, which allow additional features, such as interactivity (Ali et al., 2016). This interactivity enables the user to explore data in more detail (Perkel, 2018).

The open-source software R and the R-package Shiny allow to program such interactive graphics, which can be displayed within a web browser. These so-called Shiny-apps have increasingly gained relevance in research (Chiu et al., 2017; Di Filippo et al., 2019; Ekiz et al., 2020) and have also been used in education with promising results (Fawcett, 2018; Hanč et al., 2020; Potter et al., 2016). Jia et al. (2022) mention 470 available biological web applications developed with R-Shiny, but academic teachers might want to have individual applications related to their specific teaching topic. According to Wishart (2019) “a great way to demonstrate data to students […] is by extending use of the digital learning environment.” He found that interactive graphics build with R-Shiny can be a way to achieve this.

To master any digital learning environment, the educational staff needs to get trained in this field. However, as described by Carey and Papin (2018), many biologists are only trained in experimental techniques and have little computational literacy. Morton et al. (2019) mention that only few medical doctors have the ability to code necessary softare, too. As already stated by Fulton (1988) training the use of technology is one important factor that determines whether teachers use technology in class or not, but difficulties in teaching academic staff to create digital contents has also been described, e.g. by Hannon (2008) or Salmon and Wright (2014).

One possibility to provide educators training can be achieved through workshops. In this paper, a pilot online workshop for training academic staff to implement interactive graphics for their courses using the statistical programming environment R and the R-package Shiny. A report about a ‘workshop on graphics and visualization eduction’, which took place in 1999, lists knowledge required for those who program interactive graphics for education (Cunningham, 1999). However, requirements for the setting of the workshop itself were not reported and the R-package Shiny was not yet available at that time. Therefore, the main goal of our study accompanying the workshop was to identify relevant aspects that need to be addressed when teaching academics staff to program interactive graphics for their courses. Besides, the impact on teacher’s practice and their opinion whether interactive graphics are helpful for themselves and for their students is queried by pre- and post-workshop questionnaires.

In the following subsections, we expatiate on current knowledge about training technology to faculty members by other faculty members (as was the case in our pilot workshop), staff or students, on workshops as a means for professional development, and on additional specific research questions of our study.

### Technology-Related Faculty Development

As the integration of technology in classrooms has increased, faculty development programs on technology have become more relevant, and have already been an object of research 20 years ago (Kitano et al., 1998). Belt and Lowenthal (2020) presented a literature review on faculty development research and identified “staff-teaching-faculty” as the most common approach in teaching faculty the use of technology in class. In this format, technology skilled staff conduce workshops for teachers. Although their technical expertise allows them to provide great insights into technological possibilities, they usually lack teaching experience and are not able to contextualize teaching with technology. However, research in this field suggests that pedagogy must be considered when designing faculty development to successful influence teaching practice (Belt & Lowenthal, 2020). Therefore, “faculty-teaching-faculty” might have the biggest potential on influencing teachers’ practice. Here, technology experienced faculty serve as “technology facilitators or ambassadors” to less technology experienced teachers (Belt & Lowenthal, 2020). Another approach, “students-teaching-faculty” is less common, but can also be found in higher education, frequently in form of a reverse-mentorship (Augustiniene & Ciuciulkiene., 2013). Baran (2016) for example studied technology mentoring as a form of professional development model and concluded that it has “the potential to promote the adoption and dissemination of technology integration practice throughout higher education”. Regarding the delivery method, Belt and Lowenthal (2020) summarized that most workshops and programs are of short duration and mostly preferred to be delivered in person. Still, online professional development is expanding. Some advantages are control of pace, flexibility, and eased access to resources (Wynants & Dennis, 2018). Moreover, larger groups of faculty can access to online programs. Hybrid programmes, which combine online and in-person sessions, are less common. They lead to longer-lasting faculty development, which create a greater commitment to faculty (Belt & Lowenthal, 2020).

### Relevance of Workshops in Teachers’ Professional Development

We chose the format of a workshop to train academic staff in implementing interactive graphics as workshops have been evaluated as a very promising tool in teaching academic staff technological skills. However, it is part of our study to identify the usefulness of workshops for the specific topic of teaching academic staff to implement interactive graphics. The term workshop is used to describe a “usually brief intensive educational program for a relatively small group of people that focuses especially on techniques and skills in a particular field” (https://www.merriam-webster.com/dictionary/workshop). Georgina and Olson (2008) did a survey on more than 1000 faculty members of higher education regarding their perception towards technology training. They yielded that most faculty members agreed that learning “new computer-based technologies” could be achieved the best in small groups instructed by a trainer. Diaz et al. (2009) explicitly mentioned workshops as a reasonable tool for faculty development in technology. According to Rust (1998) from the Oxford Centre for Staff and Learning Development, workshops are a common tool for “staff and educational development […] in higher education around the world.” Workshops do not only serve to transmit knowledge. As stated by Ørngreen and Levinsen (2017), a workshop can also be used as a research methodology, when it is designed to teach participants something related to their specific interest and to answer at the same time a research question. Even though different perspectives exist, all workshops share some basic features, such as active participation and an expected outcome (Ørngreen & Levinsen, 2017). Usually, the expected outcome of teachers’ workshop is the improvement of teachers’ skills.

This can also lead to an improvement of students’ achievement since it has been suggested that both; teachers’ skills and students’ achievements; are deeply linked. (Darling-Hammond, 2000; Rice, 2003). Literature review revealed that students in higher education can benefit from teaching technologies (Concannon et al., 2005), yet some academics remain reluctant to adopt these. A lack of training has been discussed in diverse educational studies as one of the barriers to the adaption of these technologies (Abdal-Haqq, 1995; Bingimlas, 2009; Brownell & Tanner, 2012; Kurt & Ciftci, 2012; Pajo & Wallace, 2001). Schneckenberg (2009) further discussed a lack of interest in technology amongst academic staff as a reason for a slow integration of learning technologies. In the past years, there have been some studies regarding workshops to improve teachers’ technology skills. For example, Lai (2010) discussed the positive reaction of schoolteachers towards an interactive whiteboard training (IWB). After the workshop, all teachers valued the importance of IWB training workshops and recognized the potential of using IWB in class. Furthermore Liu et al. (2011) demonstrated that a computing workshop for teachers can significantly increase their confidence level in computing technologies. Although the research done in this field has added knowledge to the teaching literature, most of them centre on the learners’ satisfaction and the immediate workshop outcome rather than on the impact of those workshops on the teaching practice (Stes et al., 2010). Watson & Sottile (2010) for example studied the long-term effect of a workshop to train the integration of internet in class. He revealed that after years the workshop still had positive impact on teachers’ self-efficiency level. These studies have in common that they all analyse the effects of training educators in already implemented computer-based teaching modalities. We only found one reference to a similar workshop as ours, however, this author did not evaluate his workshop scientifically (Morota, 2020). We are not aware of previous study that has been conducted to identify relevant aspects that must be addressed in a workshop when teaching academic staff with little technological background to implement own interactive graphics. Therefore, we conducted a questionnaire based study alongside to our workshop to identify relevant issues that should be considered in future workshops.

### Research Questions

For the present work, academic staff at our university were offered to participate in an online workshop to learn how to program interactive graphics as a digital teaching tool. Such interactive graphics were already used at this university and found broad acceptance amongst teachers and students. Previous studies regarding the usage of this type of interactive graphic in education have also shown a broad acceptance amongst students (Fawcett, 2018; Potter et al., 2016). Since a workshop for academic teachers little skilled in R-programming to learn how to develop interactive R-Shiny-applications hasn’t been performed before, we also wanted to use this workshop to identify points how such a workshop could be improved. Better knowing the specific requirements for such a workshop could help us and others to host future workshops. Besides the main aim of our study, i.e. to identify requirements for future workshops on interactive graphics (e.g. the need for additional training in R programming or for specific course material), we aimed to answer the following additional questions in our study: (1) Is academic staff willing to learn programming techniques, in which they are mostly unfamiliar with, to provide digital teaching media for their students? (2) Does gender, age or previous knowledge in this field have an influence on the willingness to learn how to implement interactive graphics? (3) Does a workshop in teaching technology influence teachers’ opinion on digital media and promote a further use of the taught teaching tool? And in the light of the current COVID-19 pandemic 4) does this pandemic additionally influence the opinion towards the digitalization process of higher education?

Age and gender were included since some studies report on age and gender gaps in STEM areas to which computer sciences belong to (Atmatzidou & Demetriadis, 2016; García-Holgado et al., 2020).

We think, if workshop participants are not sufficiently prepared after the event, there is little chance that they really use interactive graphics in their courses. While an R-Shiny online workshop is quickly implemented it is not given that participants will transfer what they have learned to their teaching activities. Answering therefore the above research questions would be of great value for us and others to design future R-Shiny workshops for academic staff, and to achieve that participants are better prepared and willing to develop and use R-Shiny or even other tools in their courses.

## Methods

### Local Setting

Academic staff from different institutes of the university this research was handled were invited to participate at our workshop. The university employs about 80 professors, 20 lecturers, and 250 other staff member also involved in teaching. Occupying staff from various field of medicine, biology and natural sciences, this university constitutes an ideal environment for our research on interactive graphics. In 2005, the university established an e-learning consulting centre to promote the usage of digital teaching technologies. Additionally, in January 2019, the university launched the project ‘DigiStep’ to advance its digitalization process. The departments of physics, general radiology, chemistry, zoology, botany as well as the institute for animal breeding and genetics are partners of this project. In addition to online learning modules and case studies, video material and lectures as well as lecture recordings are used so that e-learning concepts such as blended learning and inverted classrooms can be implemented. Furthermore, the zoological exercises, in which dissections are carried out on dead animals, will be changed with the project, thereby significantly reducing the number of animals used. Our working group contributed to the ‘DigiStep’ project by implementing interactive graphics for several lectures in selected fields of medicine and natural sciences during the winter term 2019/2020.

### Program of the Online Workshop

The workshop was imparted using the online-conference tool Microsoft Teams, due to the distant rules during the COVID-19 pandemic, but also because an online communication platform is an ideal tool when teaching technology skills such as R programming. This platform not only allows to communicate per video but also to share desktop screens and files among the workshop organizer and the participants. The program of this two-day workshop included, at the first day, a presentation of previous experiences using interactive graphics as a teaching device at our university and an introduction to the statistic software R (R Core Team, 2020). The central part of the workshop consisted of teaching the functionality of the R-package Shiny (version 1.4.0). This package is based on R and allows to convert R scripts into visually appealing Shiny applications within a web browser. After day one of the workshop, participants received several R scripts as templates and other study material to create different interactive applications for their classes and self-study material. Next, participants were given one week to develop ideas, concepts, or sketches for interactive graphics for teaching propose in their own courses. At the second workshop day, we discussed these ideas and implementation possibilities.

### Study Design and Participants

Workshop participants were asked to answer an online questionnaire before and after the workshop. The study was approved by the data security office of the university, and participants took part under informed consent. The questionnaires were created using the platform Google forms and were also approved by the data security office.

Since our main research aim was to identify aspects that need to be addressed in future workshops on interactive graphics (e.g. the need for additional training in R programming), we asked on our questionnaires questions on the previous programming knowledge and experiences with digital teaching tools, and after the workshop questions on how the workshop has helped participants to implement concepts for interactive graphics. Additional items were added to the questionnaires to study our side questions such as on the influence of gender and age on the intention to implement own interactive graphics.

No personal identifiable information except for gender and age was captured and all data was stored anonymously. In total, n = 25 members of the academic staff (professors and lecturers) completed the first questionnaire and n = 15 of them also completed the second questionnaire.

The questionnaires included two participant characteristics (gender and age), open field, multiple choice and 5-point Likert scale questions ranging from 1 (strongly disagree) to 5 (strongly agree).

Item of the questionnaire submitted before the workshop are provided in Table 1. Participants were asked before the first workshop day about their programming skills and their knowledge in R. We also asked participants about their opinion towards digital media in teaching and especially interactives graphics as a teaching tool. Furthermore, we included some questions to evaluate the participant’s perception towards the importance of the COVID-19 crisis in the digitalization process in higher education**.**

**Table 1:** Questionnaire distributed to workshop participants before the first workshop day

Item	Answer options
1. Age	Open field
2. Gender	female, male, no information
3. Have you already used digital media for your class (video-conferences tools such as Microsoft-Teams and Zoom are not meant)?	Multiple choice: Video/audio-files, smartphone application, CASUS (case-based learning system), virtual microscope, Interactive graphics, podcasts, others, none
4. For how much time (in percent) is it reasonable to use digital teaching tool in higher education?	Open field
5. I find the increasing use of digital teaching media in higher education for students …	5 Point-Likert-scale question ranging from 1(negative) to 5 (positive)
6. I find the increasing use of digital teaching media in higher education for lecturers…	
7. Interactive graphics facilitate teaching	5 Point-Likert-scale question ranging from 1(strongly disagree) to 5 (strongly agree)
8. Interactive graphics facilitate learning	
9. I’m experienced in programming	Yes or no If yes, please specify
10. I have knowledge in using R	5 Point-Likert-scale question ranging from 1(strongly disagree) to 5 (strongly agree)
11. I have already used interactive graphics before	Multiple choice: on webpages (e.g. online journals) In my class
12. The invention of the letterpress in the fifteenth century reduced in my opinion the need for traditional face-to-face class	5 Point-Likert-scale question ranging from 1(strongly disagree) to 5 (strongly agree)
13. The invention of the internet in the twentieth century will fundamentally promote a permanent shift into digital teaching in higher education	
14. The COVID-19 pandemic will fundamentally promote a permanent shift into digital teaching in higher education	

The second questionnaire (Table 2) was completed by the participants after the second workshop day. Members of the academic staff were questioned if they can imagine using such interactive graphics for their teaching purpose and if they feel able to program such graphics after the workshop session. We also included a question to evaluate the workshop’s effect on their opinion towards digital teaching media.

**Table 2:** Questionnaire distributed to the workshop participants after the second workshop day

Item	Answer options
1. Age	Open field
2. Gender	Female, male, no information
3. Interactive graphics facilitate teaching	5 Point-Likert-scale question ranging from1(strongly disagree) to 5 (strongly agree)
4. Interactive graphics facilitate learning	
5. I can imagine using interactive graphics in my classes in the future	
6. I can imagine programming interactive graphics for my classes in the future	
7. The workshop promoted my interest for using digital teaching media	
8. Further comments and ideas	Open field

### Data Analysis

Answer distribution of all questions were analysed descriptively, separated by gender, age groups and by prior programming skills. Age distributions were described by means +/− SD as well as medians, minima and maxima per group. Nominal and ordinal data were described by absolute and relative frequencies. Differences between groups were further analysed using Fisher’s exact test (categorically scaled answers) or the Mann–Whitney *U* test (ordinal scaled answers). Wilcoxon signed-rank test was used to compare ordinal answers from the 1st and 2nd questionnaire. Correlations between these answers were determined using Kendall’s correlation coefficient τ. The significance level was set to α = 0.05 for all tests. Analyses were performed using again the software R (V 4.0.2, www.r-project.org).

## Results

### Workshop Participants and Prior Programming Knowledge

Workshop participants were members of the academic staff from natural sciences and the medical field. Among all participants, 80% (20/25) were females and 20% (5/25) were males. Age distribution (mean +/− standard deviation years) was 40.7 years +/− 10.5 (minimum: 26, maximum: 61 years).

Most participants (68%, 17/25), had no previous programming skills. Only a few had some knowledge in HTML (2/25) or Python (1/25) programming. Furthermore, 20% (5/25) participants had prior experience in R. The statement ‘I am fully experienced in R’ was stronly disagreed by 60% (15/25) of workshop participants. Only 3 participants agreed or stronly agreed to be experienced in R (Supplementary Table S1).

### Pre-Workshop Questionnaire: Opinion on Digital Teaching Media and on Helpfulness of Interactive Graphics

We found that most members of the participants have already used digital teaching media before the workshop. Mostly used media specified by the participants were video/audio-files (72%; 18/25) and a case-based learning system, called CASUS (24%; 6/25). Furthermore, 20% (5/25) have never used any digital teaching media before. Most of the participants (9/25) found that digital media should take up to 50% of the time in class. The rest of the participants would rather employ less time in class using digital media. Only 3/25 responders found it accurate to spent more than half of the time in class using digital devices to teach.

The mean time in percentage to spend with digital teaching technologies rated by all participants was 41.2%. Analysing these opinions separately by age we found out, that younger participants (less than 40 years old) rated for slightly less time using educational technologies in class than the older participants (older than 40 years old).

Asking about their opinion towards the effect of digital media in teaching, 48% (12/25) strongly agreed the statement ‘I find the increasing use of digital media in higher education to be positive’, both for academic staff and students. Comparing their perception on the benefits of digital media in higher education, we found that members of the academic staff rated its effect slightly more positive on students than for themselves: 20% (5/25) had a neutral perception on the benefit for members of the academic staff and 8% (2/24) disagreed to the statement digital media would be positive for academic staff in teaching. In comparison: only 4% (1/25) disagreed the statement related to students benefits of digital teaching media.

Regarding interactive graphics, 95.8% (23/24) participants found that they facilitate students to learn study content and 91.7% (22/24) find interactive graphics to be beneficial for academic staff to teach this content. In total, 40% (10/25) participants have already used interactive graphics before the workshop, 6 of them in their own class and 4 on webpages. The remaining 15 participants did not provide an answer to this question. We found no significant difference in the opinion towards the usage of digital teaching media between the age groups less than forty and over forty years old (Supplementary Table S1) nor between female and male (Supplementary Table S3) or previous programming experience (Supplementary Table S5).

### Post Workshop Questionnaire: Effects of the Workshop

**Fig. 1:**
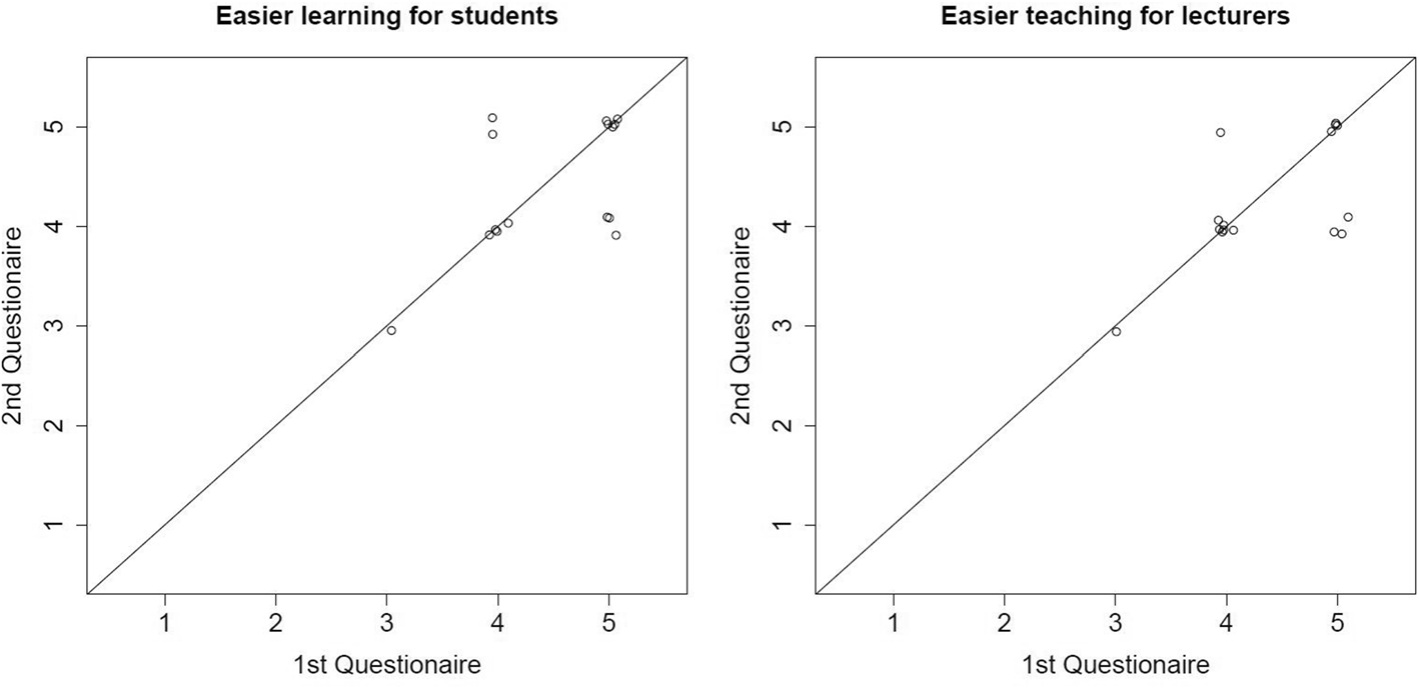
Comparison of the influence of the invention of letterpress, the internet or the occurrence of the COVID-19 pandemic on the development of digital teaching in higher education. *Note.* Levels of agreement are from 1 (no agreement) to 5 (strong agreement)

**Fig. 2:**
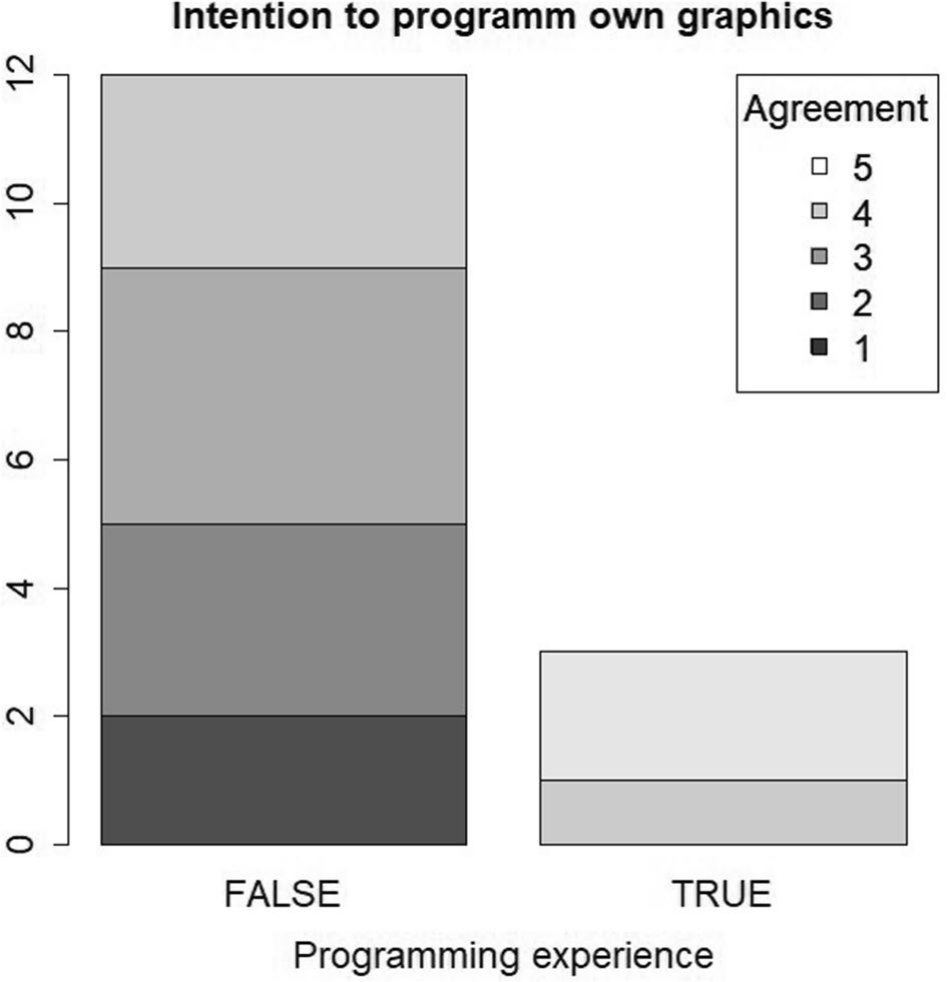
Overall high agreement to the statements that interactive graphics are positive for students (left) and lecturer (right) did little change during the workshop. *Note.* X-axis shows agreement before workshop, y-axis agreement after workshop

The second questionnaire, distributed at the end of the workshop, revealed that the positive opinion towards interactives graphics as teaching and learning tool remained unchanged (Fig. 1). There was no significant location shift in the strength of agreement (easier learning for students: *p* = 0.77; easier learning for academics staff: *p* = 0.42), although answers were only moderately correlated between 1st and 2nd questionnaire (Kendall’s τ = 0.44, *p* = 0.08 and τ = 0.57, *p* = 0.02). Most participants (85.7%, 12/14) affirm to be interested in using interactive graphics in their course, but only those who had previous programming skills (20%, 3/15) had intention to program a graphic on their own (Fig. 2, Table 3). The significance of the latter effect (*p* = 0.018) would not survive a multiple testing correction but it shows a tendency. We also asked members of the academic staff about the impact of this online workshop on their opinion towards digital teaching. According to this, 73.3% (11/15) agreed to the statement ‘the online workshop had a positive impact on my opinion towards digital teaching’ and 26.7% (4/15) had a neutral perception to this statement. Again, we found no significant difference in the opinion of the workshop participants by age (Supplementary Table S7) and gender (Supplementary Table S8).

**Table 3:** Results from the second questionnaire, divided according to previous programming experiences

Item	Levels	All participants (n = 15)	No programming experience (n = 12)	Programming experience (n = 3)	*p* Value
1. Age	M +/− SD Median (Min, Max)	38.7 +/− 12.2 34 (26,6)	40.3 +/− 13.1 36 (26, 61)	32.7 +/− 4.2 34 (28, 36)	0.47
2. Gender	Female male	12 (80%) 3 (20%)	10 (83.3%) 2 (16.7%)	2 (66.7%) 1 (33.3%)	0.52
3. Interactive graphics facilitate learning	Strongly disagree Disagree Neither… nor Agree Strongly agree	0 (0%) 0 (0%) 1 (6.7%) 7(46.7%) 7 (46.7%)	0 (0%) 0 (0%) 1 (8.3%) 6 (50%) 5 (41.7%)	0 (0%) 0 (0%) 0 (0%) 1 (33.3%) 2 (66.7%)	0.47
4. Interactive graphics facilitate teaching	Strongly disagree Disagree Neither… nor Agree Strongly agree	0 (0%) 0 (0%) 1 (6.7%) 9 (60%) 5 (33.3%)	0 (0%) 0 (0%) 1 (8.3%) 7 (58.3%) 4 (33.3%)	0 (0%) 0 (0%) 0 (33.3%) 2 (66.7%) 1 (33.3%)	0.93
5. Use interactive graphics for course	Strongly disagree Disagree Neither… nor Agree Strongly agree	0 (0%) 0 (0%) 2 (14.3%) 5(35.7%) 7 (50%)	0 (0%) 0 (0%) 2 (18.2%) 4 (36.4%) 5 (45.5%)	0 (0%) 0 (0%) 0 (0%) 1 (33.3%) 2 (66.7%)	0.49
6. Program 7. interactive graphics for course	Strongly disagree Disagree Neither… nor Agree Strongly agree	2 (13.3%) 3 (20%) 4 (26.7%) 4 (26.7%) 2 (20%)	2 (16.7%) 3 (25%) 4 (33.3%) 3 (25%) 0 (0%)	0 (0%) 0 (0%) 0 (0%) 1 (33.3%) 2 (66.7%)	0.018
8. Workshop 9. positive impact on opinion for digital teaching	Strongly disagree Disagree Neither… nor Agree Strongly agree	0 (0%) 0 (0%) 4 (26.7%) 3 (20%) 8 (53.3%)	0 (0%) 0 (0%) 5 (41.7%) 2 (16.7%) 5 (41.7%)	0 (0%) 0 (0%) 0 (0%) 1 (33.3%) 2 (66.7%)	0.31

### Influence of Letterpress, Internet and COVID-19 Pandemic on Development of Digital Teaching

**Fig. 3:**
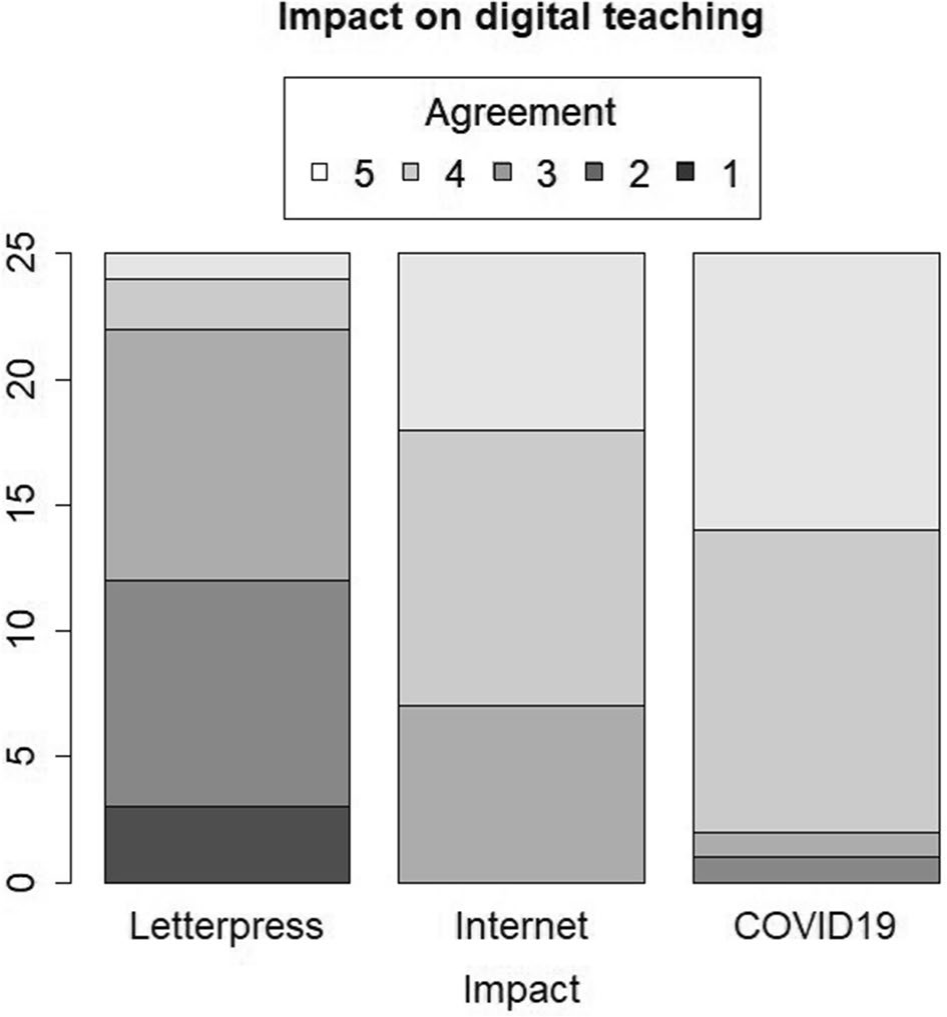
After the workshop, only participants with prior programming skills agreed to have the intention to program interactive graphics by themselves

Members of the academic staff agreed for the pandemic crisis to promote further digitalization in higher education (Fig. 3). In total, 92% (23/25) agreed or strongly agreed to the statement ‘The COVID-19 pandemic crisis will lead fundamentally to a permanent shift into digitisation in higher education’. 72% (18/25) participants agreed or strongly agreed for the invention of the internet to cause a permanent shift into digitalization in higher education. There was significantly less agreement to the statement that the invention of letterpress in the fifteenth century had a strong effect on the necessity of on-site lectures (letterpress versus internet: *p* < 0.01, letterpress versus COVID-19: *p* < 0.01). The effect of internet versus the effect of COVID-19 in digital teaching was rated as significantly different: *p* = 0.04 (Table 4). We found no significant difference towards that opinion regarding age (Supplementary Table S2), gender (Supplementary Table S4) and previous programming skills (Supplementary Table S6).

**Table 4:** Results from first questionnaire regarding the influence of letter press, internet and COVID19 pandemic on development of digital teaching

Item	Levels	All participants
The invention of the letterpress in the fifteenth century reduced in my opinion the need for traditional face-to-face class	Strongly disagree Disagree Neither… nor Agree Strongly agree	3 (12%) 9 (36%) 10 (40%) 2 (8%) 1 (4%)
The invention of the internet in the twentieth century will fundamentally promote a permanent shift into digital teaching in higher education	Strongly disagree Disagree Neither… nor Agree Strongly agree	0 (0%) 0 (0%) 7 (28%) 11 (44%) 7 (28%)
The COVID-19 pandemic will fundamentally promote a permanent shift into digital teaching in higher education	Strongly disagree Disagree Neither… nor Agree Strongly agree	0 (0%) 1 (4%) 1 (4%) 12 (48%) 11 (44%)

### Examples of Implemented Concepts Submitted by the Workshop Participants

After the first workshop day, participants were encouraged to send us their own ideas, drafts or concepts of interactive graphics, they want to employ in their courses. In total, 7 participants made submissions, which ranged from mere topic identification, to handwritten sketches and fully programmed Shiny applications. In the second workshop session, we discussed implementation possibilities with all participants. In the following the development of two Shiny application for teaching purpose are described.

#### Interactive Graphic Showing Bioavailability of Drugs for Different Types of Administration

**Fig. 4:**
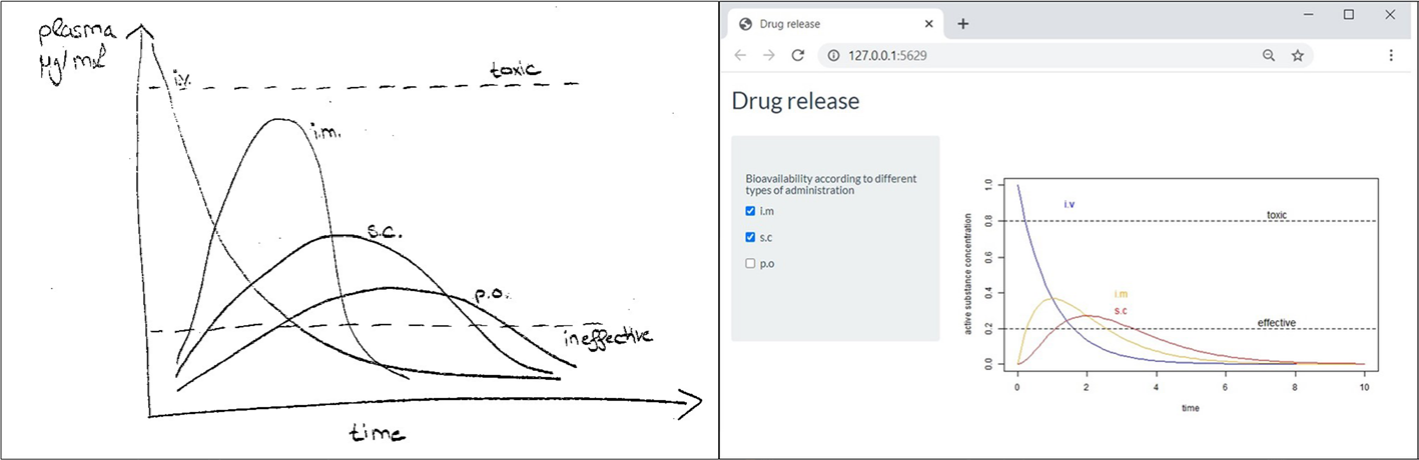
Sketch from a workshop participant for an interactive graphic in a pharmacology lecture (left) and implementation as interactive graphics with checkboxes as regulator elements (right)

Figure 4 is usually used as static graphic in the lecture of general pharmacology at our university. It is intended to give an overview of how important the chosen administration of a drug can be in terms of bioavailability and drug level (Chasseaud & Taylor, 1974). The message behind this figure is simple, but fundamental and important for a first understanding of pharmacokinetics. It is the first figure shown in the lecture and since it can be confusing due to multiple curves, it was the idea to transform the figure into an interactive graphic. This should give students a better overview, as the individual curves in the Shiny application can now be viewed in a more differentiated way by individual selection and give students the opportunity to try things out to consolidate a first understanding of pharmacokinetics. The lecturer involved in the development of this application descripted her first experience using R and Shiny as follows: ‘After the idea was developed and the first workshop took place, I could implement the idea in small steps. Since the program always showed you the errors, I was able to convert the basic structures of my idea into the Shiny app according to the trail-and-error principle.’

#### Interactive Graphic Showing Temperature-Dependent Sex Determination in Reptiles

**Fig. 5:**
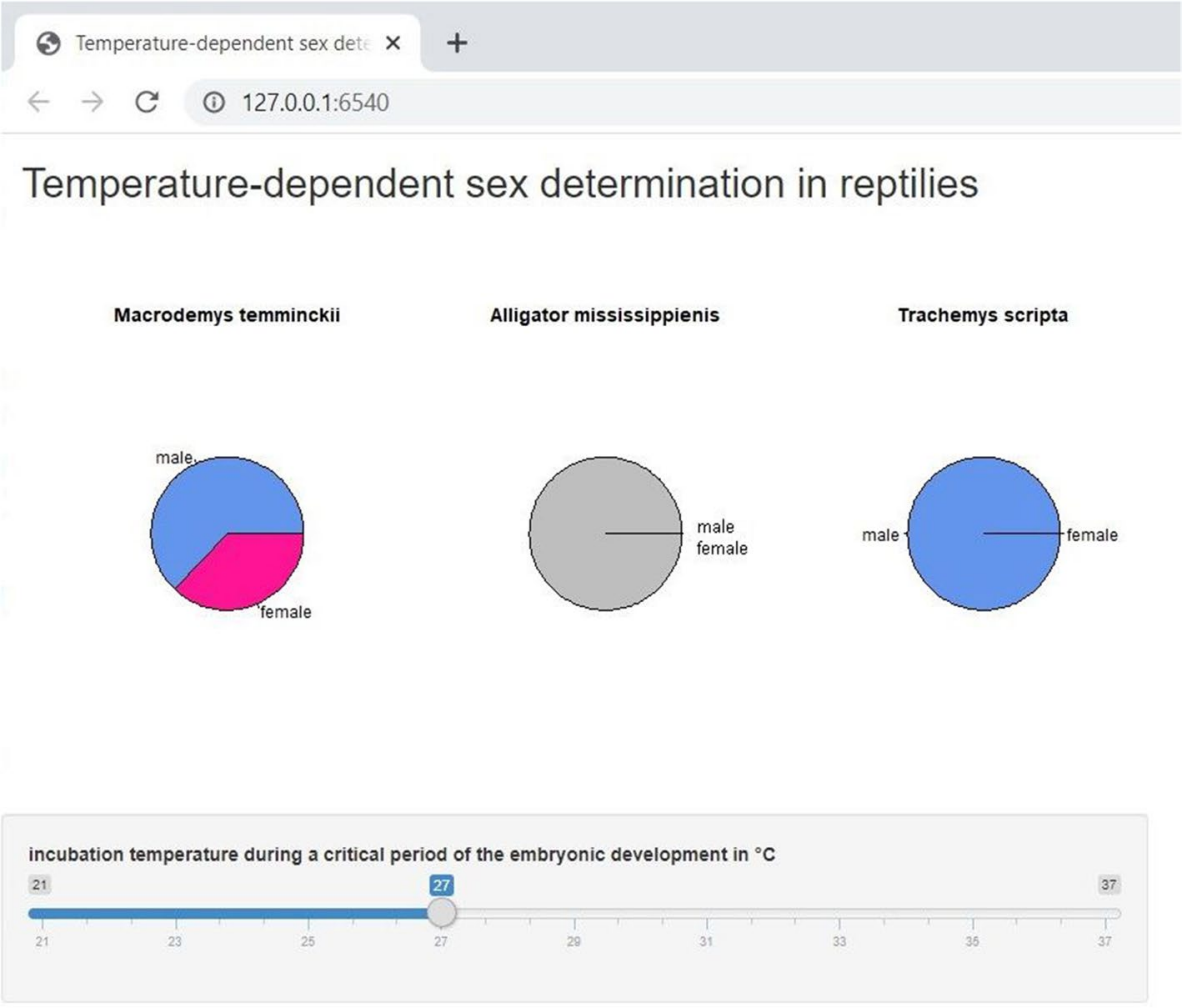
Screenshot of interactive graphic for zoology module. *Note.* The temperature-dependent sex determination for different reptile species is illustrated by pie-charts. Through the slider users can manipulate the incubation temperature. The outcoming proportion of female (pink) and male animals (blue) is shown by the pie charts. A grey coloured pie-chart appears if no information is available or no descendants are hatched at the given temperature. (Color figure online)

In some reptiles, the gender of the offspring is triggered by the incubation temperature during a critical period of the embryonic development (Mitchell et al., 2006). Thereby, three patterns exist. In the first pattern, males were produced at cooler temperatures than females. In the second pattern, females were produced at lower temperatures than males. In the third pattern, males were produced at intermediate temperatures whereas females were produced at both extremes, below and above the intermediate range. Within this study, a static line plot of a textbook (Gilbert 2000) was transferred to an interactive graphic. Here the user can regulate the temperature over a temperature slider and, thereby, the effect of incubation temperature on the sex ratio of the respective reptile species will be plotted in a pie chart (Fig. 5). Moreover, by plotting several species, the different pattern of sex determination can be additionally visualized.

Since the interactive graphic links simultaneously the movement of the temperature button to the sex proportion in the pie charts, the user can recognize on the first look and experience very fast and intuitive the mechanisms without extensive explanation, which would be necessary by using the static line plot.

The lecturer involved in the development of this application descripted her first experience with Shiny as follows: ‘After the first day of the workshop, I got interested to implement an interactive graphic to my lecture. Since I had prior experience with R and due to the guiding of the workshop organisations it was quite easy to implement this graphic by myself. But of course, it takes more time than just using static graphics from the textbook. However, for the visualisation of dynamic processes interactive graphics are very well suited and can help to simplify complex relationships.’

## Discussion

### Workshops as a Meaningful Tool to Teach Academic Staff to Implement Interactive Graphics

Scientific data has become more complex in many scientific disciplines and static graphics are often not suitable to represent the information inherent to these data. E.g., in the biomedical disciplines, a large amount of high-dimensional or big data is collected in experiments of studies. Interactive graphics, e.g. implemented by means of the R-package Shiny appear to be more suitable for these kind of data. Interactive graphics contain regulator elements which allow to manipulate the data behind the graphic, and the subsequent effects are displayed immediately. This synchronicity has been stated as one important aspect of interactivity (Liu & Shrum, 2002). Teacher can benefit from using interactive graphics since they can explain gradually the content of the graphic.

The present study investigates how academic staff can be taught via a workshop to implement their own interactive graphics, and what would be necessary for future workshop. The workshop was led by one technology experienced faculty (KJ) and a doctoral student (PL), combining the faculty-teaching-faculty and student-teaching-faculty approaches described by Belt and Lowenthal (2020). We believe to have combined the advantages of these two approaches. While the student can provide classroom experience and contextualize students understanding, faculty can encourage colleagues to actively participate, combat resistance (Belt & Lowenthal, 2020) and connect teaching practice with technology.

In a personal feedback some participants also outlined the need for a second workshop or further support. These findings are in line with Watsons’ (2006) study results regarding the effects of a 5-day workshop to train teachers the internet usage in class. He proved that teachers had a higher level of self-efficiency after attending to online courses in addition to the workshop, than those who just participated to the workshop. Still, most faculty development workshops are short (Belt & Lowenthal, 2020).

Specifically, we found that the online format of our workshop was very useful since the organizers and participants could share screen and files among each other. Thus, participants could for example share their programming code and the instructors could give direct feedback on it.

### Aspects to be Addressed in Future Workshops

Our questionnaire based study yielded that most of the participants showed strong interest in using interactive graphics for their courses. The participants also rated interactive graphics in general as helpful for the lecturers as well as for students as an additional learning tool. Prior to the workshop, more than 90% of the participants agreed or strongly agreed to the statements that interactive graphics facilitate teaching and learning (Supplementary Table S1). However, although we included basics of graphic programming in R, only 25% of the participants with little or no prior programming background expressed that they still feel enough prepared to implement interactive graphics on their own. These were significantly less participants compared to the subgroup with prior programming knowledge (*p* = 0.018). Furthermore, only 7 out of 25 participants submitted ideas for own interactive graphics before the second workshop day. Consequently, we derive as necessary items for future workshops.

- additional training in R programming for the subgroup with no prior programming skills, and
- to provide more templates for own interactive graphics and to spend more time with participants to elaborate on these templates together.

Especially to develop own interactive graphics by the participants as described in Sect. 3.5 emerged as an important part of the workshop.

Age and gender aspects, in contrast, did not emerge as issues that need specifically addressed in such a workshop. No significant correlations with age or gender and the answers of the first and second questionnaire were detected (Supplementary Tables S1, S3, S7 and S8).

Further results of our study indicate that academic staff were open-minded to expand their programming skills and to learn how to employ a new digital teaching tool. Over 70% of the participants agreed or strongly agreed that the workshop had a positive impact on their opinion for digital teaching (Supplementary Table S7). Between the different age groups, we found no evidence for different perception towards digital teaching tools or the willingness to use such. Interestingly, older workshop participants had a higher interest in investing more time using digital teaching devices in class than the younger ones. This finding contradicts the theory of a ‘digital generation gap’ (Buckingham, 2006; Kelty, 2002). This term refers to an inter-generational, inequality in the usage of technology and suggest that older generation have a harder time adapting to new technologies. The average age of our participants was 40.7 years. Our participants therefore cannot be categorized as ‘digital natives’ (Jones et al., 2010). A lack of interest or motivation among academic staff towards technology as stated by Lazar (2015) or Schneckenberg (2009) was not confirmed in this study. Most of the members of the academic staff, who participated at our workshop, had no previous programming nor basic R skills, but they got fully engaged to the topic and had own ideas and drafts of interactive graphics for their teaching purpose.

Another reason why participants may remain reluctant to program their own interactive graphics might be due to the time-consuming development process. Some studies have already thematised that digital teaching added workload to lecturers (Bright, 2012; Waycott et al., 2010; Young & McSporran, 2004). Lai (2010) further stated that some teachers expressed doubts regarding the time and energy investment linked to the design of interactive features for lesson. However, there has been growing evidence for the value of interactivity in enhancing learning experience (Evans & Gibbons, 2007; Teoh & Neo, 2007; Zhang et al., 2006).

Although our pilot workshop had a limited small sample size, the participants represent a diverse range of age, institutes and specialisation. They are all from different academic backgrounds, where programming is not a common skill. We recommend providing further coaching and support for teaching staff to get actively involved in the development of digital teaching tools.

Another solution to bring interactive graphics into academic courses would be to let interested lecturer provide schemes and to discuss with them which interactive elements could be established. Next, a programming expert could implement the interactive graphic. The two examples presented in Sect. 3.5. show that this can be a successful approach. However, additional time must be scheduled for the programmer, then.

### Effect of the COVID-19 Pandemic on Digitalization Process of Higher Education

More recently the COVID-19 pandemic has evidenced the importance of technology trained teachers and academic staff. When the global lockdown and quarantine policies forced educational institution worldwide to close, teachers had to move from traditional face-to-face to online education (Bao, 2020; Murphy, 2020).

As a side aspect, we studied the participant’s opinion towards the impact of the COVID-19 crisis on education. More than 90% of the participants assigned the COVID-19 crisis an important role in advancing digitalization in higher education (agreement or strong agreement). It´s impact was rated by the respondents to be even higher than the invention of the internet at the end of the twentieth century. Only 72% agreed or strongly agreed that the invention of the internet will promote a permanent shift into digital teaching. This results somehow contradicts educational pre-COVID-19 studies, which assign the internet a crucial role in digitalization in higher education (Cookson, 2000; Pittinsky, 2003; Stošić & Stošić, 2015; Twigg, 2002). In a ‘future of the Internet’ survey conducted in 2012, digital stakeholders were asked to imagine education in 2020. 60% agreed with the scenario “By 2020, higher education will be quite different from the way it is today. There will be mass adoption of teleconferencing and distance learning […]. There will be a transition to "hybrid" classes that combine online learning components with less-frequent on-campus, in-person class meetings.” (Anderson et al., 2012). The COVID-19-crisis has demonstrated that this future vision did not became fully true. In the current situation universities often simply offer text-file and lecture captures to the students, providing rather “remote” than online education (Gardner, 2020), raising the question of how online teaching technologies can get promoted and successfully implemented in the future. The COVID-19 crisis has shown the urgent need for lecturers to get training and coaching in online teaching technologies (Houlden & Veletsianos, 2020). Gardner (2020) went further and declared the COVID-19 crisis as a “wake-up call” for universities to think of a “long-term digital strategy”.

## Conclusions

The evaluation of this pilot workshop shows that there is much interest among academic lecturers from diverse fields to use interactive graphics in their courses and that they rate these tools as helpful for themselves but also for the students. Since only academics staff with prior programming skills see themselves as sufficiently prepared to implement interactive graphics on their own, additional training in R programming must be scheduled for upcoming workshops, and more efforts must be made to work with the participants on their own ideas for interactive graphics. Alternatively, one could consider to provide separate workshops for participants with and without programming skills.

## Supplementary Information

Below is the link to the electronic supplementary material.

Supplementary file1 (PDF 649 kb)

## Data Availability

The data that support the findings of this study are openly available in Mendeley at http://dx.doi.org/10.17632/4zsggk9swp.1 (https://doi.org/10.17632/4zsggk9swp.1).
