# Supplementary material for: Teaching Academic Staff to Implement Interactive Graphics for Their Courses
Source: Technol Knowl Learn. 2023 May 31:1–22. Online ahead of print. doi: 10.1007/s10758-023-09652-y (PMC10230133; doi:10.1007/s10758-023-09652-y)
Supplement: Supplementary file 1 — Supplementary file1 (PDF 649 kb) [file 10758_2023_9652_MOESM1_ESM.pdf]

Supplementary Table S1

Answers from the first questionnaire divided by the age-groups ≤ 40 years and > 40 years old

Item		Levels	NA	All participants	<= 40 years (n=13)	Older 40 years (n=12)	p-value
1.	Age (years)	M +/- SD		40.7 +/- 10.5	32.5 +/- 4.9	49.7+/- 6.9	
		Median (Min, Max)		40 (26, 61)	33 (26, 40)	49 (41,61)	
2.	Gender	Female		20 (80%)	12 (92%)	4 (33.3%)	.16
		Male		5 (20%)	1 (8%)	8 (66.7%)	
3.	Used digital teaching media before	no		5 (20%)	2 (15.4%)	3 (25%)	*
		CASUS		6 (24%)	4 (30.8%)	2 (16.7%)	
		Interact. Graph.		5 (20%)	2(15.4%)	3 (25%)	
		Apps		1 (4%)	0 (0%)	1 (8.3%)	
		Video/Audio		18(72%)	10 (7%)	8 (66.7%)	
		Other		5 (20%)	3 (23.1%)	2 (16.7%)	
4.	Percentage in courses	Mean +/- SD		41.2 +/- 19.4	38.5 +/- 10.88	44.2+/-26.0	.61
		Median (Min, Max)		40 (10, 100)	40 (20, 50)	50 (10,100)	
5.	Digital media positive for studs.	Strongly disagree		0 (0%)	0 (0%)	0(0%)	.13
		Disagree		1 (4%)	0 (0%)	1(8.3%)	
		Neither... nor		3 (12%)	1 (7.7%)	2(16.7%)	
		Agree		9 (36%)	4 (30.8%)	5(41.7)	
		Strongly agree		12 (48%)	8 (61.5%)	4(33.3%)	
6.	Digital media positive for lect.	Strongly disagree		0(0%)	0 (0%)	0(0%)	.43
		Disagree		2(8%)	0 (0%)	2(16.7%)	
		Neither... nor		5(20%)	3 (23.1%)	2(16,7%)	
		Agree		6(24%)	3 (23.1%)	3(25.0%)	
		Strongly agree		12(48%)	7 (53.8%)	5(41.7%)	
7.	Interactive graphics facilitate teaching	Strongly disagree	1	0(0%)	0 (0%)	0 (0%)	.62
		Disagree		0(0%)	0 (0%)	0 (0%)	
		Neither... nor		2(8,3%)	0 (0%)	2(18.2%)	
		Agree		12(50%)	9 (69.2%)	3(27.3%)	
		Strongly agree		10(41,7%)	4(30.8%)	6(54.5%)	
8.	Interactive graphics facilitate learning	Strongly disagree	1	0(0%)	0 (0%)	0 (0%)	.10
		Disagree		0(0%)	0 (0%)	0 (0%)	
		Neither... nor		1(4,2%)	3 (23.1%)	1 (9.1%)	
		Agree		11(45,8%)	3 (23.1%)	2 (18.2%)	
		Strongly agree		12(50%)	7 (53.8%)	8 (72.7%)	
9.	Previous programming skills	None		17(68%)	8 (61.5%)	9(75.0%)	.46
		In R		5(20%)	4 (30.8%)	1(8.3%)	
		Other		3(12%)	1 (7.7%)	2(16.7%)	
10.	Strongly experienced in R	Strongly disagree		15(60%)	7 (53.8%)	8 (66.7%)	.73
		Disagree		4(16%)	3 (23.1%)	1(8.3%)	
		Neither... nor		3(12%)	2 (15.4%)	1(8.3%)	
		Agree		1(4%)	0 (0%)	1(8.3%)	
		Strongly agree		2(8%)	1 (7.7%	1(8.3%)	
11.	Used interact. graph. before	On webpage		4(16%)	2 (15.4%)	2 (16.67%)	1.00
		in my course		6(24%)	3 (23.01%)	3 (0.25%)	
		no		15 (60%)	7 (53.8%)	9 (75%)	

Note. Questions are abbreviated here and translated from German to English

* No p-value calculated due to multiple choice answers

Supplementary Table S2

Answers of the first questionnaire regarding the influence of letterpress, internet and COVID19 pandemic on development of digital teaching divided by the age-groups less than forty and mayor than forty years old

Item	Levels	All participants	<= 40 years (n=13)	Older 40 years (n=12)	p-value
12. Invention of the Letterpress reduced need for traditional face-to-face teaching	Strongly disagree	3 (12%)	2 (15.4%)	1 (8.3%)	.18
	Disagree	9 (36%)	6 (46.2%)	3 (25%)	
	Neither... nor	10 (40%)	4 (30.8%)	6 (50%)	
	Agree	2 (8%)	1 (7.7%)	1 (8.3%)	
	Strongly agree	1 (4%)	0 (0%)	1 (8.3%)	
13. Invention of the internet will promote a permanent shift into digital teaching in higher education	Strongly disagree	0 (0%)	0 (0%)	0 (0%)	1.00
	Disagree	0 (0%)	0 (0%)	0 (0%)	
	Neither... nor	7 (28%)	4 (30.8%)	3 (25%)	
	Agree	11 (44%)	5(38.5%)	6 (50%)	
	Strongly agree	7 (28%)	4 (30-8%)	3 (25%)	
14. Corona pandemic will promote a permanent shift into digital teaching in higher education	Strongly disagree	0 (0%)	0 (0%)	0 (0%)	.54
	Disagree	1 (4%)	0 (0%)	1 (8.3%)	
	Neither... nor	1 (4%)	0 (0%)	1 (8.3%)	
	Agree	12 (48%)	7 (53.8%)	5 (41.7%)	
	Strongly agree	11 (44%)	6 (46.2%)	5 (41.7%)	

Supplementary Table S3

Answers from the first questionnaire by gender

Item		Levels	NA	All participants	female (n=20)	male (n=5)	p-value
1.	Age (years)	M +/- SD		40.7 +/- 10.5	39.5 +/- 10,7	45.8 +/- 9.04	.17
		Median (Min, Max)		40 (26, 61)	38.5 (26, 61)	45 (34, 57)	
2.	Gender	Female		20 (80%)	20	5	
		Male		5 (20%)			
3.	Used digital teaching media before	no		5 (20%)	5 (25%)	0 (0%)	a)
		CASUS		6 (24%)	5 (25%)	1 (20%)	
		Interact. graphics		5 (20%)	0 (0%	0 (0%)	
		Apps		1 (4%)	0 (0%)	1 (20%)	
		Video/Audio		18(72%)	14 (70%)	4 (80%)	
		Other		5(20%)	4 (20%)	1 (20%)	
4.	Percentage in courses	Mean +/- SD		41.2 +/- 19.4	40 +/- 19.5	46 +/- 20.4	.51
		Median (Min, Max)		40 (10, 100)	40 (10,100)	50 (25, 70)	
5.	Digital media positive for studs.	Strongly disagree		0 (0%)	0 (0%)	0 (0%)	.21
		Disagree		1 (4%)	0 (0%)	1 (20%)	
		Neither... nor		3 (12%)	3 (15%)	0 (0%)	
		Agree		9 (36%)	6 (30%)	3 (60%)	
		Strongly agree		12 (48%)	11 (55%)	1 (20%)	
6.	Digital media positive for lect.	Strongly disagree		0(0%)	0 (0%)	0 (0%)	.20
		Disagree		2(8%)	1 (5%)	1 (20%)	
		Neither... nor		5(20%)	4 (20%)	1 (20%)	
		Agree		6(24%)	4 (20%)	2 (40%)	
		Strongly agree		12(48%)	11 (55%)	1 (20%)	
7.	Interactive graphics facilitate teaching	Strongly disagree	1	0(0%)	0 (0%)	0 (0%)	.32
		Disagree		0(0%)	0 (0%)	0 (0%)	
		Neither... nor		2(8,3%)	2 (10.5%)	0 (0%)	
		Agree		12 (50%)	10 (52.6%)	2 (40%)	
		Strongly agree		10 (41,7%)	7 (36.8%)	3 (60%)	
8.	Interactive graphics facilitate learning	Strongly disagree	1	0(0%)	0 (0%)	0 (0%)	.15
		Disagree		0(0%)	0 (0%)	0 (0%)	
		Neither... nor		1(4,2%)	1 (5.3%)	0 (0%)	
		Agree		11(45,8%)	10 (52.6%)	1 (20%)	
		Strongly agree		12(50%)	8 (42.6%)	4 (80%)	
9.	Previous programming skills	None		17(68%)	14 (70%)	3 (60%)	.13
		In R		5(20%)	5 (25%)	0 (0%)	
		Other		3(12%)	1 (5%)	2 (40%)	
10.	Strongly experienced in R	Strongly disagree		15(60%)	12 (60%)	3 (60%)	.97
		Disagree		4(16%)	3 (15%)	1 (20%)	
		Neither... nor		3(12%)	3 (15%)	0 (0%)	
		Agree		1(4%)	1 (5%)	0 (0%)	
		Strongly agree		2(8%)	1 (5%)	1 (0%)	
11.	Used interactives graphics before	On webpage		4(16%)	2 (10%)	2 (40%)	.29
		in my course		6 (24%)	5 (25%)	1 (20%)	
		no		15 (60%)	13 (65%)	2 (40%)	

Note. Questions are abbreviated here and translated from German to English. a) No p-value calculated due to multiple choice answers

Supplementary Table S4

Answers of the first questionnaire regarding the influence of letterpress, internet and COVID19 pandemic on development of digital teaching divided by gender.

Item	Levels	All participants	female (n=20)	male (n=5)	p-value
12. Invention of the Letterpress reduced need for traditional face-to-face teaching	Strongly disagree	3 (12%)	3 (15%)	0 (0%)	.08
	Disagree	9 (36%)	8 (40%)	1 (20%)	
	Neither... nor	10 (40%)	8 (40%)	2 (40%)	
	Agree	2 (8%)	0 (0%)	2 (40%)	
	Strongly agree	1 (4%)	1 (5%)	0 (0%)	
13. Invention of the internet will promote a permanent shift into digital teaching in higher education	Strongly disagree	0 (0%)	0 (0%)	0 (0%)	.54
	Disagree	0 (0%)	0 (0%)	0 (0%)	
	Neither... nor	7 (28%)	7 (35%)	0 (0%)	
	Agree	11 (44%)	7 (35%)	4 (80%)	
	Strongly agree	7 (28%)	6 (30%)	1 (20%)	
14. Corona pandemic will promote a permanent shift into digital teaching in higher education	Strongly disagree	0 (0%)	0 (0%)	0 (0%)	.38
	Disagree	1 (4%)	1 (5%)	0 (0%)	
	Neither... nor	1 (4%)	1 (5%)	0 (0%)	
	Agree	12 (48%)	10 (50%)	2 (40%)	
	Strongly agree	11 (44%)	8 (40%)	3 (60%)	

Supplementary Table S5

Answers of the first questionnaire divided by programming experience

Item		Levels	NA	All participants	No programming experience (n=17)	Programming experience (n=8)	p-value
1.	Age (years)	M +/- SD<br>Median (Min, Max)		40.7 +/- 10.5<br>40 (26, 61)	41.9 +/- 11.7<br>41 (26, 61)	38.3 +/- 7.5<br>37.5 (28, 52)	1.00
2.	Gender	Female<br>Male		20 (80%)<br>5 (20%)	14 (82.4%)<br>3 (17.6%)	6 (75%)<br>2 (25%)	.52
3.	Used digital teaching media before	no<br>CASUS<br>Interact. graphics<br>Apps<br>Video/Audio<br>Other		5/25 (20%)<br>6/25 (24%)<br>5/ 25 (20%)<br>1 /25 (4%)<br>18 /25(72%)<br>5/25 (20%)	4 (23.5%)<br>5 (29.4%)<br>4 (23.5%)<br>1 (5.9%)<br>12 (70.6%)<br>2 (11.8%)	1 (12.5%)<br>1 (12.5%)<br>1 (12.5%)<br>0 (0%)<br>6 (75%)<br>2 (25%)	*
4.	Percentage in courses	Mean +/- SD<br>Median (Min, Max)		41.2 +/- 19.4<br>40 (10, 100)	40.9 +/- 22.6<br>40 (10, 100)	41.9 +/- 11.3<br>50 (25, 50)	.65
5.	Digital media positive for studs.	Strongly disagree<br>Disagree<br>Neither... nor<br>Agree<br>Strongly agree		0 (0%)<br>1 (4%)<br>3 (12%)<br>9 (36%)<br>12 (48%)	0 (0%)<br>1 (5.9%)<br>2 (11.8%)<br>6 (35.3%)<br>8 (47.1%)	0 (0%)<br>0 (0%)<br>1 (12.5%)<br>3 (12.5%)<br>4 (50%)	.82
6.	Digital media positive for lect.	Strongly disagree<br>Disagree<br>Neither... nor<br>Agree<br>Strongly agree		0(0%)<br>2(8%)<br>5(20%)<br>6(24%)<br>12(48%)	0 (0%)<br>1 (5.9%)<br>5 (29.4%)<br>3 (29.4%)<br>8 (47.1%)	0 (0%)<br>1 (12.5%)<br>0 (0%)<br>3 (37.5%)<br>4 (50%)	.68
7.	Interactive graphics facilitate teaching	Strongly disagree<br>Disagree<br>Neither... nor<br>Agree<br>Strongly agree	1	0(0%)<br>0(0%)<br>2(8,3%)<br>12(50%)<br>10(41,7%)	0 (0%)<br>0 (0%)<br>1 (6.2%)<br>8 (50%)<br>7 (43.8%)	0 (0%)<br>0 (0%)<br>1 (12.5%)<br>4 (50%)<br>3 (37.5%)	.70
8.	Interactive graphics facilitate learning	Strongly disagree<br>Disagree<br>Neither... nor<br>Agree<br>Strongly agree	1	0(0%)<br>0(0%)<br>1(4,2%)<br>11(45,8%)<br>12(50%)	0 (0%)<br>0 (0%)<br>1 (6.2%)<br>6 (37.5%)<br>9 (56.2%)	0 (0%)<br>0 (0%)<br>0 (0%)<br>5(62.5%)<br>3 (37.5%)	.53
9.	Previous programming skills	None<br>In R<br>Other		17(68%)<br>5(20%)<br>3(12%)	17 (100%)<br>0 (0%)<br>0 (0%)	0 (0%)<br>5(62.5%)<br>3 (37.5%)	
10.	Strongly experienced in R	Strongly disagree<br>Disagree<br>Neither... nor<br>Agree<br>Strongly agree		15(60%)<br>4(16%)<br>3(12%)<br>1(4%)<br>2(8%)	14 (82.4%)<br>1 (5.9%)<br>1 (5.9%)<br>0 (0%)<br>1 (5.9%)	1 (12.5%)<br>3 (37.5%)<br>2 (25%)<br>1(12.5%)<br>1 (12.5%)	.002
11.	Used interactive graphics before	On webpage<br>in my course<br>no		4(16%)<br>6(24%)<br>15 (60%)	2 (11.8%)<br>5 (29.4%)<br>10 (58.8%)	2 (25%)<br>1 (12.5%)<br>5 (62.5%)	.50

***** *No p-value calculated due to multiple choice answers*

Supplementary Table S6

Answers of the first questionnaire regarding the influence of letter press, internet and COVID19 pandemic on development of digital teaching divided by programming experience

Item	Levels	All participants	No programming experience (n=17)	Programming experience (n=8)	p-value
12. Invention of the Letterpress reduced need for traditional face-to-face teaching	Strongly disagree	3 (12%)	2 (11.8%)	1 (12.5%)	.76
	Disagree	9 (36%)	6 (35.3%)	3 (37.5%)	
	Neither... nor	10 (40%)	8 (47.1%)	2 (25%)	
	Agree	2 (8%)	1 (5.9%)	1(12.5%)	
	Strongly agree	1 (4%)	0 (0%)	1 (12.5%)	
13. Invention of the internet will promote a permanent shift into digital teaching in higher education	Strongly disagree	0 (0%)	0 (0%)	0 (0%)	.28
	Disagree	0 (0%)	0 (0%)	0 (0%)	
	Neither... nor	7 (28%)	6 (35.3%)	1 (12.5%)	
	Agree	11 (44%)	7 (41.2%)	4 (50%)	
	Strongly agree	7(28%)	4 (23.5%)	3 (37.5%)	
14. Corona pandemic will promote a permanent shift into digital teaching in higher education	Strongly disagree	0 (0%)	0 (0%)	0 (0%)	.85
	Disagree	1 (4%)	1 (5.9%)	0 (0%)	
	Neither... nor	1 (4%)	0 (0%)	1 (12.5%)	
	Agree	12 (48%)	9 (52.9%)	3 (37.5%)	
	Strongly agree	11 (44%)	7 (41.2%)	4 (50%)	

Supplementary Table S7

Answers of the second questionnaire divided by the age-groups ≤ 40 years and > 40 years old

Item		Levels	NA	All participants	<=40 years (n= 10)	Older 40 years (n=5)	p-value
1.	Age	M +/- SD		38.7 +/- 12.2	31.1 +/- 4.4	53.8 +/- 6.5	
		Median (Min, Max)		34 (26, 61)	30.5 (26, 38)	56 (44, 61)	
2.	Gender	Female		12 (80%)	9 (90%)	3 (60%)	.24
		male		3 (20%)	1 (10%)	2 (40%)	
3.	Interactive graphics facilitate learning	Strongly disagree		0 (0%)	0 (0%)	0 (0%)	.84
		Disagree		0 (0%)	0 (0%)	0 (0%)	
		Neither... nor		1 (6.7%)	0 (0%)	1 (20%)	
		Agree		7(46.7%)	6 (60%)	1 (20%)	
		Strongly agree		7 (46.7%)	4 (40%)	3 (60%)	
4.	Interactive graphics facilitate teaching	Strongly disagree		0 (0%)	0 (0%)	0 (0%)	.44
		Disagree		0 (0%)	0 (0%)	0 (0%)	
		Neither... nor		1 (6.7%)	0 (0%)	1 (20%)	
		Agree		9 (60%)	8 (40%)	1 (20%)	
		Strongly agree		5 (33.3%)	2 (50%)	3 (60%)	
5.	Use interactive graphics for course	Strongly disagree	1	0 (0%)	0 (0%)	0 (0%)	.46
		Disagree		0 (0%)	0 (0%)	0 (0%)	
		Neither... nor		2 (14.3%)	2 (22.2%)	0 (0%)	
		Agree		5(35.7%)	3 (33.3%)	2 (40%)	
		Strongly agree		7 (50%)	4 (44.4%)	3 (60%)	
6.	Program interactive graphics for course	Strongly disagree		1 (6.7%)	1 (10%)	0 (0%)	.35
		Disagree		3(20%)	2 (20%)	1 (20%)	
		Neither... nor		4 (26.7%)	2 (20%)	2 (40%)	
		Agree		4 (26.7%)	3 (30%)	1 (20%)	
		Strongly agree		3 (20%)	2 (20%)	1 (20%)	
7.	Workshop positive impact on opinion for digital teaching	Strongly disagree		0 (0%)	0 (0%)	0 (0%)	.47
		Disagree		0 (0%)	0 (0%)	0 (0%)	
		Neither... nor		4 (26.7%)	4 (40%)	0 (0%)	
		Agree		3 (20%)	2 (20%)	1 (20%)	
		Strongly agree		8 (53.3%)	4 (40%)	4 (80%)	

Supplementary Table S8

Answers of the second questionnaire divided by gender

Item		Levels	NA	All participants	female (n=12)	male (n=3)	p-value
1.	Age	M +/- SD		38.73+/- 12.2	37.1 +/- 12.2	45 +/- 11.5	
		Median (Min, Max)		34 (26,6)	33.5 (26, 61)	44 (34, 57)	
2.	Gender	Female		12 (80%)			.25
		male		3 (20%)			
3.	Interactive graphics facilitate learning	Strongly disagree		0 (0%)	0 (0%)	0 (0%)	.47
		Disagree		0 (0%)	0 (0%)	0 (0%)	
		Neither... nor		1 (6.7%)	1 (8.3%)	0 (0%)	
		Agree		7(46.7%)	6 (50%)	1 (33.3%)	
		Strongly agree		7 (46.7%)	5(41.7%)	2 (66.7%)	
4.	Interactive graphics facilitate teaching	Strongly disagree		0 (0%)	0 (0%)	0 (0%)	.93
		Disagree		0 (0%)	0 (0%)	0 (0%)	
		Neither... nor		1 (6.7%)	1 (8.3%)	0 (0%)	
		Agree		9 (60%)	7 (58.3%)	2 (66.7%)	
		Strongly agree		5 (33.3%)	4 (33.3%)	1 (33.3%)	
5.	Use interactive graphics for course	Strongly disagree	1	0 (0%)	0 (0%)	0 (0%)	.49
		Disagree		0 (0%)	0 (0%)	0 (0%)	
		Neither... nor		2 (14.3%)	2 (18.2%)	0 (0%)	
		Agree		5(35.7%)	4 (36.4%)	1 (33.3%)	
		Strongly agree		7 (50%)	5 (45.5%)	2 (66.7%)	
6.	Program interactive graphics for course	Strongly disagree		1 (6.7%)	1 (8.3%)	0 (0%)	.34
		Disagree		3(20%)	2 (16.7%)	0 (0%)	
		Neither... nor		4 (26.7%)	4 (33.3%)	1 (33.3%)	
		Agree		4 (26.7%)	3 (25%)	1 (33.3%)	
		Strongly agree		3 (20%)	2 (16.7%)	1 (33.3%)	
7.	Workshop positive impact on opinion for digital teaching	Strongly disagree		0 (0%)	0 (0%)	0 (0%)	.82
		Disagree		0 (0%)	0 (0%)	0 (0%)	
		Neither... nor		4 (26.7%)	4 (33.3%)	0 (0%)	
		Agree		3 (20%)	2 (16.7%)	1 (33.3%)	
		Strongly agree		8 (53.3%)	6 (50%)	4 (66.7%)	
